# Matrix Effects on the Stability and Antioxidant Activity of Red Cabbage Anthocyanins under Simulated Gastrointestinal Digestion

**DOI:** 10.1155/2014/365738

**Published:** 2014-01-19

**Authors:** Anna Podsędek, Małgorzata Redzynia, Elżbieta Klewicka, Maria Koziołkiewicz

**Affiliations:** ^1^Institute of Technical Biochemistry, Faculty of Biotechnology and Food Sciences, Lodz University of Technology, Stefanowskiego 4/10, 90-924 Łódź, Poland; ^2^Institute of Fermentation Technology and Microbiology, Faculty of Biotechnology and Food Sciences, Lodz University of Technology, Wólczańska 171/173, 90-924 Łódź, Poland

## Abstract

Red cabbage is, among different vegetables, one of the major sources of anthocyanins. In the present study an in vitro digestion method has been used to assay the influence of the physiological conditions in the stomach and small intestine, as well as faecal microflora on anthocyanins stability in red cabbage and anthocyanin-rich extract. The recovery of anthocyanins during in vitro gastrointestinal digestion was strongly influenced by food matrix. The results showed that other constituents present in cabbage enhanced the stability of anthocyanins during the digestion. The amount of anthocyanins (HPLC method) and antioxidant capacity (ABTS and FRAP assays) strongly decreased after pancreatic-bile digestion in both matrices but total phenolics content (Folin-Ciocalteu assay) in these digestions was higher than in initial samples. Incubation with human faecal microflora caused further decline in anthocyanins content. The results obtained suggest that intact anthocyanins in gastric and products of their decomposition in small and large intestine may be mainly responsible for the antioxidant activity and other physiological effects after consumption of red cabbage.

## 1. Introduction

*Brassica* vegetables are consumed by people all over the world and represent an important part of human diet. Common types of these vegetables used for food preparation include different genuses of cabbage (white, green, and red), broccoli, brussels sprouts, cauliflower, and kale. They are of great interest in nutrition and medicine because of their potent protective effects on human health. Published reports link a high intake of *Brassica* vegetables with reduced risk of age-related chronic illnesses such as cardiovascular diseases and several types of cancer [1–3].

Red cabbage (*Brassica oleracea* var. *capitata rubra*) is known for its protective role against cardiac and hepatic oxidative stress in rats fed an atherogenic diet [4], hypocholesterolemic activity in tumour-bearing mice [5], neuroprotective activity in N-methyl-D-aspartate-treated rats [6, 7], paraquat-induced oxidative stress in rats [8], nephroprotective activity in diabetic rats [9], and hepatoprotective activity in carbon tetrachloride-treated rats [10]. Moreover, in vitro polyphenol extract from red cabbage influenced properties of erythrocyte membrane with high concentration of cholesterol [11] and demonstrated high antioxidant activity [12–17]. Among the substances that seem to be responsible for those properties are phenolic compounds, especially anthocyanins. The profile of red cabbage anthocyanins is very complex, and so far up to 36 different forms of the compounds have been found in this vegetable [18]. Red cabbage anthocyanins are cyanidin 3-diglucoside-5-glucoside derivatives highly conjugated with sugars (glucose and xylose) and acyl groups (caffeoyl, *p*-coumaroyl, feruloyl, *p*-hydroxybenzoyl, sinapoyl, and oxaloyl) [18–20].

Despite the discovery of many health benefits of anthocyanins, their bioavailability is still not clear and unreliable. Previous studies have shown very low urinary recovery of acylated anthocyanins from steamed red cabbage (<0.05%) and from raw and cooked purple carrots (<0.015%) [18, 21]. Furthermore, in human studies recovery of acylated anthocyanins was about 4-fold lower than recovery of nonacylated forms in case of red cabbage and 11–14-fold lower for purple carrot anthocyanins. According to Charron and coworkers [18] red cabbage anthocyanins were excreted in human urine in both intact and metabolised forms, such as glucuronidated anthocyanins.

The overall bioavailability process includes gastrointestinal digestion, absorption, and metabolism. Thus, in the gastrointestinal tract anthocyanins may be released from the food matrix, modified under the influence of digestive enzymes, as well as a result of pH changes. The digestive stability of different food components can be assessed by in vitro digestion method. To our knowledge, only one study reports the effect of in vitro gastrointestinal digestion on the stability of red cabbage anthocyanins. This study has been carried out on the anthocyanin concentrates produced by sorption to C18 solid phase extraction (SPE) [22]. The authors observed high anthocyanins stability in the acidic-gastric digestion conditions and low recovery of total anthocyanins (about 27%) after simulated pancreatic digestion, wherein acylated anthocyanins were more stable than nonacylated forms. Low recovery (1.9%) of anthocyanins in the “serum available” fraction from the in vitro digestion procedure suggested that the most of red cabbage anthocyanins are likely to reach the colon. In the large intestine anthocyanins are subjected to bacterial metabolism by colon flora and transformed mainly into 4-hydroxybenzoic acid, *p*-coumaric, and ferulic and caffeic acids [23].

In the present study, we examined the stability of red cabbage anthocyanins during the in vitro gastrointestinal digestion and incubation with human faecal suspension. Because the food matrix affects bioavailability of bioactive compounds the experiment was carried out on (i) the raw cabbage, that is, eaten as an ingredient of various salads, and (ii) anthocyanin-rich extract bereft other vegetable components. Additionally, this is the first study which is focused on evaluating the changes in antioxidant activity of red cabbage anthocyanins after the in vitro gastric and intestinal digestion.

## 2. Materials and Methods

### 2.1. Materials and Chemicals

Red cabbage cv. Koda used for the assays was obtained from the PlantiCo Horticulture Breeding and Seed Production, Gołębiew, Ltd. (central region of Poland). Chlorogenic acid, gallic acid, 6-hydroxy-2,5,7,8-tetramethyl-chromane-2-carboxylic acid (Trolox), 2,2′-azinobis(3-ethylbenzthiazoline-6-sulfonic acid) (ABTS), 2,4,6-tris-2-pyridyl-*s*-triazine (TPTZ), potassium persulfate, vitamin K, formic acid, pancreatin, bile salts, and pepsin were delivered by Sigma-Aldrich (Steinheim, Germany). Cyanidin 3-glucoside was delivered by Extrasynthese (Genay, France). HPLC grade methanol and acetonitrile were obtained from J.T. Baker (Germany). All other chemicals were reagent grade products purchased from POCh (Gliwice, Poland). Ultra purity water was prepared in the laboratory using a Simplicity Water Purification System (Millipore, Marlborough, MA, USA).

### 2.2. Isolation of Anthocyanin-Rich Extract

Lyophilised red cabbage (10 g) was extracted twice with 500 mL and 250 mL of 70% methanol at room temperature for 30 min under shaking. The mixture was then centrifuged at 4000 rpm for 15 min, and supernatant was evaporated under reduced pressure (*T* < 40°C). The aqueous extract was adjusted to pH 7 with 0.1 M NaOH and loaded on a C18 Sep-Pak cartridge (10 g capacity, Waters Corp., Milford, MA) previously conditioned with methanol followed by water (pH 7) [24, 25]. The cartridge was washed with water (pH 7), followed by ethyl acetate to elute phenolic acids and flavonoids. The anthocyanins were eluted with methanol-HCl mixture (99.9 : 0.1 v/v) until colourless eluate was obtained. Then the eluate was evaporated under reduced pressure (*T* < 40°C) and the remaining solid was dissolved in water. The anthocyanin-rich extract was used in the digestion procedure.

### 2.3. Condition of the In Vitro Digestion Process

The digestion procedure was carried out accordingly to the method published by Gil-Izquierdo et al. [26]. Samples for the in vitro digestion process were prepared by taking 50 g of the homogenized edible parts of raw red cabbage or 60 mL of anthocyanin-rich extract and an addition of 150 or 140 mL of water, respectively. All samples were placed in a 250 mL beakers (with water jacket) equipped in three inlets allowing the introduction of pH electrode, thermometer, and dosage of biochemical agents. To simulate gastric digestion 60000 units of pepsin (EC 2.4.23.1) were added to the samples, whose pH was adjusted to 2 with 1 M HCl. The mixture was incubated with shaking at a 37°C for 2 h in the absence of light. At the end of the gastric digestion, the aliquots of samples (50 g or 50 mL) were removed for anthocyanins and antioxidant activity analysis. The residue was diluted with water (50 mL) and adjusted to pH 5 with 0.5 M NaHCO_3_. Then, after 30 min, 5 mL of a pancreatin (4 g/L)-bile salts (25 g/L) mixture was added to the samples and pH of this mixture was adjusted to 7.4 with 0.5 M NaHCO_3_. The resulting mixtures were incubated for an additional 2 h. The digestion procedure was performed in triplicate.

Red cabbage before digestion and samples removed after its gastric and pancreatic digestion were extracted twice with 70% aqueous methanol at room temperature for 30 min under shaking. The mixtures were then centrifuged at 4000 rpm for 15 min, and supernatants were evaporated under reduced pressure (*T* < 40°C). The obtained aqueous extracts, as well as the anthocyanin-rich extract and its digested samples, were analysed directly in order to determine anthocyanin profile by HPLC method, total phenols content, and the antioxidant capacity.

### 2.4. Incubation with Human Faecal Microflora

In order to evaluate the stability of anthocyanins in the presence of human faecal microbiota the aliquots (4 mL) of pancreatic-bile digests were inoculated with human faecal slurry (5 mL) inoculed 10% (v/v) and incubated in an anaerobic chamber (an atmosphere of the gas mixture was H : N : CO_2_ ratio 1 : 8 : 1 (v/v/v) for 48 hours at 37°C. After defined periods (0, 6, 12, 24, and 48 h) the samples were centrifuged at 4000 rpm for 10 min, passed through a 0.22 *μ*m filter (type Millex GS, Millipore, Carrigtwahill, Co. Corc, Ireland), and the supernatants were analyzed in order to determine anthocyanin profile by HPLC method. The faecal suspension was also incubated without the digested samples as a control for each time point. All incubations were performed in triplicate.

The faecal suspension was prepared from mixed faecal samples obtained from 14 healthy volunteers (female 10, men 4; 17–52 years). Equal amounts of faecal samples were diluted to 10% (w/w) with phosphate buffered saline (PBS, 0.1 M, pH 7.0) and homogenized in a homogenizer IUL Instruments (Merck) for 1 min. Faecal microbiota were grown on the medium with the composition (g/L): peptone (1.0), yeast extract (1.0), NaCl (0.5), K_2_PO_4_ (0.02), MgSO_4_·H_2_O (0.005), CaCl_2_·6H_2_O (0.005), NaHCO_3_ (1.0), bile salts (0.25), Tween 80 (1.0), lactose (10.0), and agar (0.2) and supplements: vitamin K (5 *μ*L/L), hemine (0.0025 g/L), and cysteine (0.25 g/L); pH of the medium was adjusted to 7.0 if necessary. Culture medium before inoculation of faecal microflora was incubated in the anaerobic chamber for 24 h to remove excess oxygen. The growth medium after degassing (5 mL) was inoculated with 10% (v/v).

### 2.5. Bacterial Enumeration

In order to assess the changes in bacterial population the samples after 48 h incubation of faecal suspension with digested anthocyanins were analysed with the use of the standard plate tests. The following groups of microorganisms were quantitatively determined on selective media: *Lactobacillus* sp., Rogosa Agar (Merck); *Bifidobacterium* sp., RB Agar [27], *Clostridium* sp., TSC Agar (Merck); *Bacteroides* sp., Schaedler Agar (BioMerieux) supplemented with 5% (v/v) sheep blood, kanamycin-vancomycin mixture (BioMerieux), and vitamin K 0.01% (w/v); *Enterococcus* sp., Bile Aesculin Agar (Merck); *Enterobacteriaceae* family, MacConkey Agar (Merck), total number of anaerobic bacteria was determined using Scheadler Agar (BioMerieux) [28].

### 2.6. Qualitative Analysis of Anthocyanins

The anthocyanin profiles of nondigested or digested raw cabbage or anthocyanin-rich extract were analyzed by HPLC Knauer system equipped with UV-Vis detector and a Eurospher-100 C18 column (25 cm × 4.6 mm; 5 *μ*m). The binary mobile phase consisted of water/formic acid (90 : 10, v/v) (solvent A) and water/acetonitrile/formic acid (40 : 50 : 10, v/v/v) (solvent B), according to Dyrby et al. [29]. The system was run with a flow rate of 1 mL/min and the following gradient program: 0 min: 88% A + 12% B, 26 min: 70% A + 30% B, 40–43 min: 0% A + 100% B, 43–48 min: 88% A + 12% B, and 48–50 min: 88% A + 12% B. Anthocyanins were detected at 520 nm and their content was expressed as cyanidin 3-glucoside equivalents. All samples before injection were passed through a 0.45 *μ*m filter (type minisartRC4, Sartorius AG, Goettingen, Germany).

### 2.7. Determination of Total Phenolic Compounds

Total phenolics content was determined using a Folin-Ciocalteu method [30]. Phenolic contents were estimated from a standard curve of gallic acid.

### 2.8. Isolation and Quantification of Anthocyanins

Anthocyanin-rich extract obtained from red cabbage was applied to column chromatography on the Sephadex LH-20 (100 cm × 2.5 cm) and eluted with 10% formic acid at a flow rate of 1 mL/min [31]. The 160 aliquots of 4 mL were collected and analyzed at 530 nm (spectrophotometer He*λ*ios *α*, Unicam, England) and then pooled into eight fractions, which were further purified by SPE (C18 Sep-Pak cartridge, 10 g capacity, Waters Corp., Milford, MA) preconditioned with methanol, followed by water. The adsorbed anthocyanins were eluted with methanol containing 1% (v/v) HCl after washing with water. The eluate was then evaporated to dryness at 30°C under reduced pressure, and the solid residue was dissolved in 3 mL of 96% ethanol. Each fraction was independently analyzed by means of HPLC (as described above) and MALDI-TOF MS.

### 2.9. Analysis by MALDI-TOF MS

To obtain structural information regarding to the anthocyanins analyzed, a MALDI-TOF MS analysis was performed using a Voyager-Elite MALDI-TOF mass spectrometer (Per-SeptiveBiosystems Inc., Framingham, MA) equipped with delayed extraction system. Typical conditions included 20 kV acceleration voltage and nitrogen laser pulse (wavelength 337 nm). High-resolution positive-ion spectra were recorded in reflector mode. 2,5-Dihydroxybenzoic acid was used as a matrix. MALDI-TOF MS analysis was carried out in the Centre of Molecular and Macromolecular Studies of the Polish Academy of Sciences in Łódź.

### 2.10. ABTS Radical Cation Scavenging Activity

ABTS^∙+^ scavenging activity was determined according to a procedure described by Re et al. [32]. 2,2′-Azinobis(3-ethylbenzthiazoline-6-sulfonic acid) radical cation (ABTS^∙+^) was produced by the mixing of 7 mM ABTS water solution and 2.45 mM potassium persulfate (final concentration) and allowing the mixture to stand before use for 12–16 h in the dark at room temperature. Stock solution was diluted with PBS (pH 7.4) until an absorbance of 0.76 (±0.02) at 734 nm and was reached. Each sample analyzed (20 *μ*L) was mixed with 1 mL of diluted ABTS^∙+^ solution and incubated at 30°C for 6 min. Afterward, the absorbance was measured at 734 nm was against a blank of PBS. Trolox (6-hydroxy-2,5,7,8-tetramethylchromane-2-carboxylic acid) was used as a standard and the capacity of free radical scavenging was expressed as *μ*moles of Trolox equivalents (TE: Trolox equivalent).

### 2.11. Ferric Reducing Antioxidant Power (FRAP) Assay

The FRAP (Ferric Reducing Antioxidant Power) assay developed by Benzie and Strain [33] was performed with some modification. Briefly, 2.7 mL of FRAP reagent, prepared freshly and warmed to 30°C, was mixed with water (0.27 mL) and the analyzed sample (0.09 mL). The FRAP reagent was prepared by mixing 2.5 mL of a 10 mM solution of 2,4,6-tri-2-pyridyl-*s*-triazine (TPTZ) in 40 mM HCl with 2.5 mL of 20 mM FeCl_3_·6H_2_O and diluting with 25 mL of 0.3 mM acetate buffer, pH 3.6. Absorbance at 593 nm was recorded after 10 min incubation of the solution at 30°C. Results were expressed as *μ*moles of Trolox equivalents (TE).

### 2.12. Statistical Analysis

The data for differences between the digestion phase and the results of microbial analysis were analyzed using one-way analysis of variance (ANOVA) and presented as mean ± standard deviations. A *P* value of ≤ 0.05 was considered significant.

## 3. Results

### 3.1. Effect of Simulated Digestion on Red Cabbage Anthocyanins Stability

The effect of in vitro gastrointestinal digestion on total phenolics determined by Folin-Ciocalteu method and anthocyanins content calculated as the sum of individuals estimated by HPLC method is presented in Table 1. The in vitro gastrointestinal digestion consisted of two sequential steps, an initial pepsin/HCl digestion for 2 h to simulate gastric conditions followed by a digestion with pancreatin-bile salts for 2.5 h to simulate small intestine conditions. The gastric treatment of raw red cabbage enhanced significantly anthocyanins content (by 62.7%), but total phenolics content increased only by 25.1%. On the other hand, after a gastric digestion of anthocyanin-rich extract increase of 16.9% in the total phenolics content was only apparent, while anthocyanins content remained unchanged. The transition of sample from the acidic gastric (pH 2) to the mild alkaline intestinal environment (pH 7.4) caused a significant decrease in the amount of anthocyanins as compared with the initial samples, wherein the higher anthocyanins stability was observed for red cabbage (67.7% recovery versus 13.2% for anthocyanin-rich extract)—Table 1. Significant anthocyanins degradation during pancreatic-bile digestion of anthocyanin-rich extract resulted in significant increase of total phenols (by 29.9%), while phenolics content of intestinal red cabbage digestion was not significantly changed. Differences between the results for red cabbage and isolated anthocyanin-rich extract suggest that amount of anthocyanins released during digestion process and their stability strongly depend on the food matrix.

**Figure 1:**
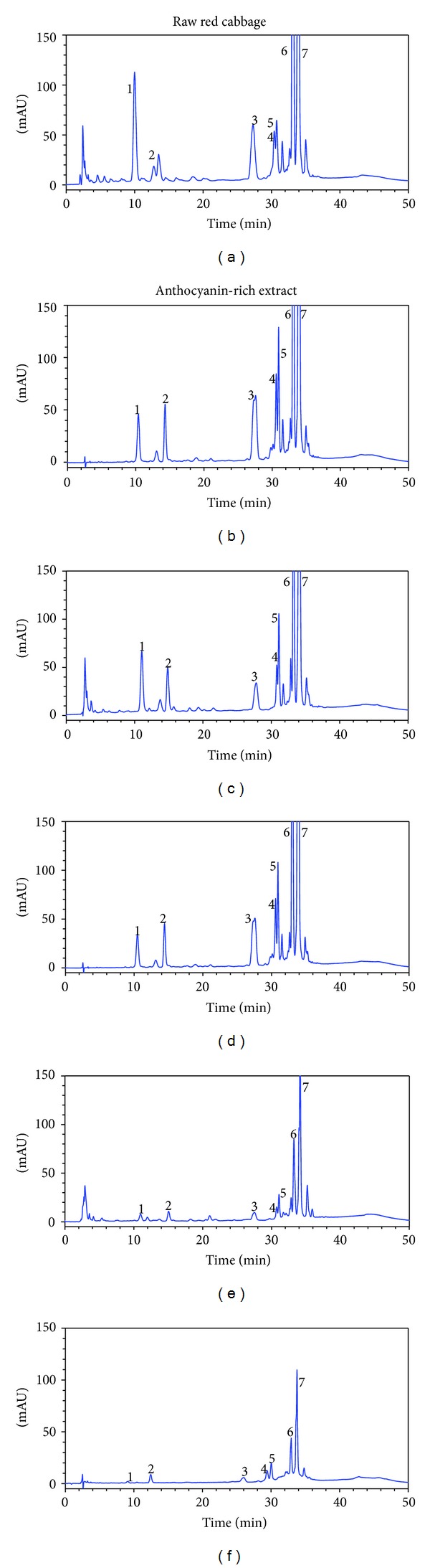
HPLC chromatograms of anthocyanins (520 nm) nondigested ((a), (b)), after pepsin digestion ((c), (d)), and after pancreatic-bile ((e), (f)) digestion. The peak numbers and assignments are given in Table 2.

Qualitative and quantitative compositions of anthocyanins in red cabbage and in anthocyanin-rich extract before and after digestion are presented in Figure 1 and Table 2. The analysis of anthocyanins in red cabbage is difficult because the reference compounds are not commercially available, so we putatively identified anthocyanins by comparison of MALDI-TOF mass spectra of isolated anthocyanins with previously reported data [18–20, 22]. Raw red cabbage extract and anthocyanin-rich extract showed very similar anthocyanin profiles. One nonacylated and twelve acylated anthocyanins were identified in both experimental materials. Peaks 2 and 3 were made up of two coeluting molecular species, while peaks 6 and 7 were made up of three coeluting anthocyanins. In addition, anthocyanins present in peaks 6 and 7 comprised 72.2% and 72.9% of total anthocyanins in red cabbage and anthocyanin-rich extract, respectively.

**Figure 2:**
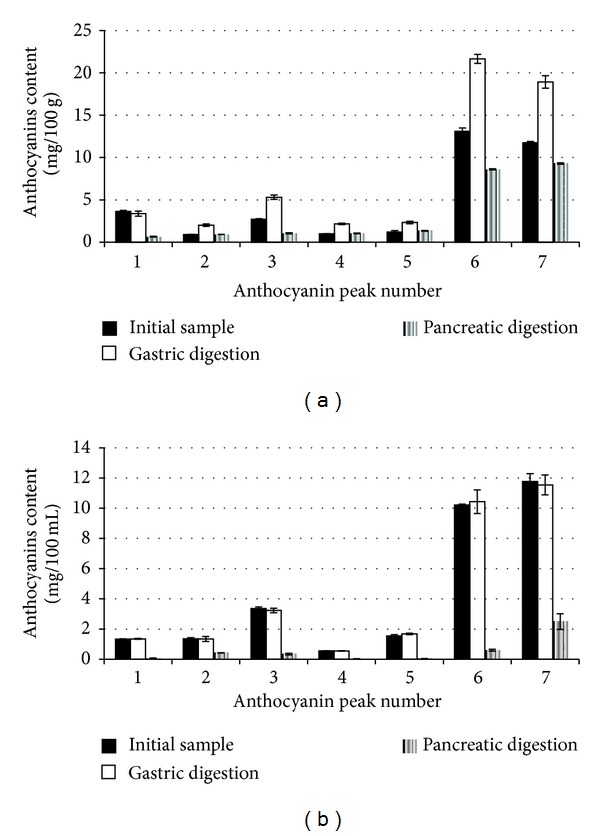
Content of anthocyanins after simulated digestion of raw red cabbage (a) and anthocyanin-rich extract (b). The anthocyanin peak numbers and assignments are given in Table 2.

The recovery of the individual anthocyanins after gastric and pancreatic-bile digestions as compared to the content in initial samples are shown in Figure 2. The digestions possessed the same major anthocyanins as the original samples. The stability of anthocyanins during the gastrointestinal digestion depended on anthocyanins structure and food matrix (whole red cabbage versus liquid anthocyanin-rich extract). The recovery of anthocyanins in red cabbage or anthocyanin-rich extract digests was reported to vary from 17.96 to 112.50% and from 2.97 to 30.88%, respectively. Among anthocyanins examined, nonacylated cyanidin 3-diglucoside-5-glucoside exhibited the lowest recovery during digestion of whole red cabbage, while in case of anthocyanin-rich extract the lowest recovery was observed for cyanidin 3-(feruloyl)(sinapoyl) triglucoside-5-glucoside. On the other hand, we observed 1.6-2.2-fold higher concentration of acylated anthocyanins after gastric digestion of red cabbage. These results suggest that acidic conditions of gastric digestion could improve the release of anthocyanins from the solid food matrix.

### 3.2. Effect of Faecal Microflora on Red Cabbage Anthocyanins

**Figure 3:**
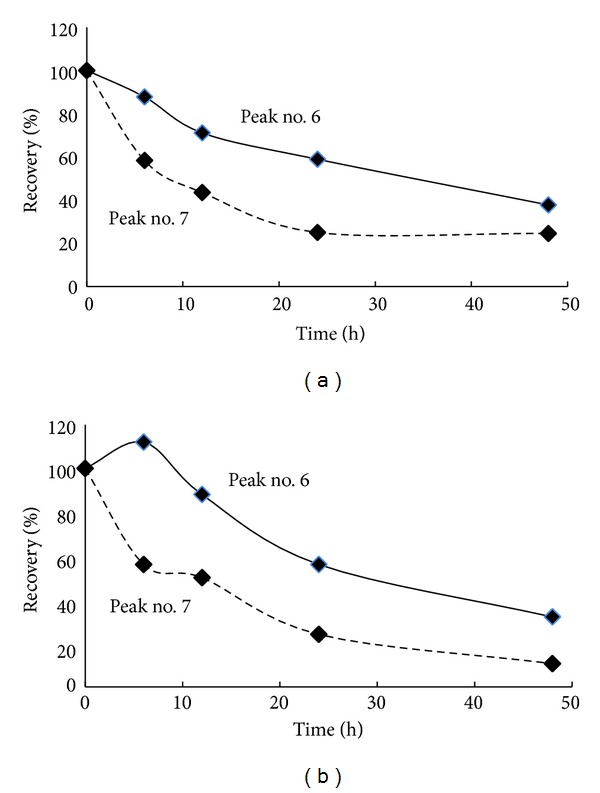
Recovery of selected anthocyanins from peaks number 6 and 7 after incubation of gastrointestinal digests with human faecal microflora for 48 h. Composition of the peaks are given in Table 2.

Food components unabsorbed in upper gastrointestinal tract enter into large intestine where they are subjected to bacterial metabolism by colon flora. In order to access the stability of red cabbage anthocyanins in the colon we incubated gastrointestinal digests with human faecal suspension for 48 h. Incubation media collected at 0, 6, 12, 24, and 48 h were analyzed using HPLC method in order to evaluate the amount of major anthocyanins which survived the gastrointestinal digestion (Figure 2). The following anthocyanins were analyzed: cyanidin 3-diglucoside-5-glucoside monoacylated with sinapic, ferulic or *p*-coumaric acids (peak number 6 in Figure 1) and cyanidin 3-diglucoside-5-glucoside diacylated with aforesaid phenolic acids (peak no. 7). The anthocyanins tested were continuously degraded during incubation—Figure 3. The recovery of monoacylated anthocyanins after 48 h incubation was calculated for red cabbage and anthocyanin-rich extract to be 38% and 32%, and diacylated anthocyanins—23% and 22%, respectively. This similar recovery in both samples may result from the use of extractable polyphenols in case of red cabbage instead suspension after digestion.

An addition of phenolic extract from digested red cabbage to culture of faecal microbiota resulted in reductions in the growth of all the tested groups of microorganisms (Table 3). The most limited was the growth of bacteria of the *Bacteroides* genus. We noticed a decrease of the bacteria number by 4.6 logarithmic units (LogU). In the case of addition of the digested anthocyanin-rich extract reduction of the growth of *Bacteroides* sp. was only 1.7 LogU. However, between these groups, there was no statistically significant difference (*P* = 0.06137). It is worth to underline reduction of the number of *Enterococcus* sp., *Enterobacteriaceae*, and *Clostridium* bacteria in both groups. The number of *Lactobacillus* sp. and *Bifidobacterium* sp. after the addition of both digests tested to the culture system was slightly decreased as compared to the control group. The *Lactobacillus* sp. and *Bifidobacterium* sp. bacteria are responsible for maintaining the homeostasis of the intestinal microbiota and drastic reduction in their numbers in the system is not desirable.

### 3.3. Influence of In Vitro Digestion on Antioxidant Capacity

Health benefits of anthocyanins are partly attributed to their antioxidant activity. The antioxidant capacities of initial and digested samples were evaluated by measurements of the free radical scavenging effect (ABTS method) and the ferric reducing antioxidant power (FRAP method). The products subjected to the digestion process have shown different initial antioxidant capacity, expressed as Trolox equivalent (TE)—Table 4. The initial TE values were threefold and twofold higher for raw red cabbage than for anthocyanin-rich extract in ABTS and FRAP assay, respectively. Thus, the higher phenolic compounds content in the case of whole vegetable corresponded to higher scavenging and reducing capacities. The antioxidant capacities of both samples after gastric digestion did not significantly differ as compared to initial samples, whereas they were significantly lower after pancreatic-bile digestion. In the intestinal digestions of red cabbage or anthocyanin-rich extract, the ABTS^∙+^ radical scavenging activities decreased by 36% and 49%, while ferric reducing activities by 40% and 50%, respectively. The reduction of these parameters could be due to some unidentified chemical transformations of phenolic compounds, especially acylated anthocyanins.

## 4. Discussion

Red cabbage (*Brassica oleracea* var. *capitata rubra*) is well known to be a rich source of anthocyanins, especially acylated anthocyanins and this vegetable exhibits high antioxidant capacity [12, 18, 34–36]. HPLC and MALDI-TOF MS analyses of red cabbage extract and anthocyanin-rich extract have shown the presence of thirteen anthocyanins (Table 2). These results are in agreement with those reported by Dyrby et al. [29], who found in red cabbage fifteen anthocyanins. On the other hand, this number is considerably smaller as compared to data reported by other authors, who found in red cabbage 2–6 nonacylated and 18–30 acylated anthocyanins [18–20, 22]. Major anthocyanins peaks (numbers 6 and 7, Figure 1) were identified as monoacylated and diacylated derivatives of cyanidin 3-diglucoside-5-glucoside. These compounds constituted about 70% of total anthocyanins in both initial samples tested. According to Charron et al. [18] aforesaid anthocyanins constituted 50% of total content of 36 identified anthocyanins.

After oral ingestion of 100–300 g of steamed red cabbage anthocyanins, both in intact and metabolised forms, have been recovered in urine in very small amounts (<0.2%) [18]. The red cabbage anthocyanins absorption was affected by their structure, because the recovery of nonacylated anthocyanins was 4-fold higher than that one of acylated anthocyanins. This low bioavailability may suggest that the majority of acylated anthocyanins remains in the gastrointestinal tract where they may exert important healthy effect. Numerous studies report the effect of in vitro gastrointestinal digestion on the stability of anthocyanin in liquid matrices, such as red wine [37], pomegranate juice [38], raspberry extract [39], isolated mulberry [40], and red cabbage anthocyanins [22]. In these studies, the high recovery (98-99%) or a slight increase in anthocyanins concentration (up to 10%) was observed during gastric digestion. In our study the recoveries of individual anthocyanins after gastric digestion of anthocyanin-rich extract were similar (96–108%, Figure 2(b)). Whereas, gastric digestion of solid matrices, such as fruit and vegetables resulted in a significant increase of anthocyanins. The amount of bioaccessible total anthocyanins after 2 h of gastric digestion increased 2.8-fold for plum [41], 1.9-fold for grape [42], and 1.6-fold for red cabbage in the present study (Table 1). Our results confirm the data cited that the conditions occurring in gastric cause a significant release of anthocyanins from food matrix. In fruits and vegetables anthocyanins can be linked to carbohydrates, proteins, and cell walls as well as to other phenolic compounds by covalent bonds, hydrogen bonding, and hydrophobic and hydrophilic interactions [43]. In addition, the differences between food matrix composition and structure seem to be very important. Compounds bound to solid food matrix are released by the action of strong acidic environmental (pH 2) and digestive enzymes which disrupt macromolecules [44]. The low pH in gastric is also a factor increasing high stability of anthocyanins, which occur as stable flavylium cations [45].

The transition from the acidic gastric to the mild alkaline intestinal environment decreased the total anthocyanin content in red cabbage and anthocyanin-rich extract by 58% and 87%, respectively. As reported in our work, the stability of anthocyanins during intestinal digestion differs strongly dependently on food matrices. Probably, other components of red cabbage protect more labile anthocyanins. Our results on the stability of anthocyanins under pancreatic-bile digestion are in agreement with those reported for juices (37–43% loss) [38, 46], red wine (62%) [37], plum (93%) [41], and grape (79%) [42]. The low recovery of anthocyanins during pancreatic digestion may be mainly due to transformation of the flavylium cation form to the chalcone and carbinol pseudobase. At pH values higher than 7, the anthocyanins are degraded depending on their structure [45]. Woodward and coworkers [47] reported that degradation of cyanidin 3-glucoside during incubation in physiological buffer (pH 7.4) over 24 h was associated with the formation of protocatechuic acid and phloroglucinol aldehyde, which accounted for 12% and 3% of the initial concentration, respectively. On the other hand, Bermúdez-Soto et al. [46] suggested that interactions with the digestive enzymes were not responsible for anthocyanins losses during in vitro digestion. In addition, the content and stability of anthocyanins included in foodstuff in the gastrointestinal tract can be influenced by other components present in meals. The codigestion of raspberry anthocyanins with ice cream, bread, breakfast cereal, or cooked minced beef caused a decrease in their content [39].

Among the 13 acylated anthocyanins indicated in this study, the highest stability was noticed for cyanidin 3-(sinapoyl)diglucoside-5-glucoside and cyanidin 3-(glycopyranosyl-sinapoyl)diglucoside-5-glucoside in the case of anthocyanin-rich extract digestion, although the cyanidin 3-diglucoside-5-glucoside monoacylated with *p*-coumaric acid, ferulic, or sinapic acid and diacylated with these acids (peaks number 6 and 7) dominated in postintestinal digestion (Figure 2(b)). McDougall et al. [22] observed that cyanidin 3-diglucoside-5-glucoside monoacylated with *p*-coumaric acid was the most stable during the pancreatic-bile digestion of isolated red cabbage anthocyanins. Cyanidin 3-diglucoside-5-glucoside and cyanidin 3-(glycopyranosyl-*p*-coumaroyl)-diglucoside-5-glucoside with cyanidin 3-(glycopyranosyl-feruloyl)diglucoside-5-glucoside were the less stable anthocyanins in digested whole red cabbage.

An application of a dialysis membrane during the pancreatin-bile digestion showed that dialysed anthocyanins, which are described as “serum-available,” represented only 1.9–5.3% of initial anthocyanins content [22, 37–39], therefore most of the anthocyanins are colon-available. On the other hand, according to Bermúdez-Soto et al. [46], the use of a dialysis membrane as a means of estimating availability for absorption has some limitations and the results cannot be truly associated with absorption in vivo. He et al. [48] postulated that faeces are a major excretion route of ingested anthocyanins. Furthermore, the authors observed that acylation of grape anthocyanins with *p*-coumaric acid increased their resistance to absorption or degradation in the gut. In physiological pH anthocyanins were degraded to benzoic acid derivatives, with the protocatechuic acid as the predominant product of cyanidin degradation [23, 49, 50]. This acid was the major product after anaerobic incubation of cyanidin and cyaniding 3-glucoside with a human faecal suspension.

Polyphenols and their derived products can also affect the intestinal ecology by accumulation in the ileal and colorectal lumen of nondigested structures, nonabsorbed components, and metabolites [51]. In the present study it is worth to underline the reduction of the number of *Enterococcus* sp., *Enterobacteriaceae*, and *Clostridium* bacteria in both groups. The bacteria included in these groups are producers of fecal enzymes (*β*-glucuronidase, azoreductase, nitroreductase, *β*-glucosidase, *β*-galactosidase, and 7-*α*-dehydroxylase) which are involved in conversions of endogenous toxins and genotoxic compounds [52]. The reduction in the number of bacteria with high faecal enzymes activity may be responsible for lower risk of toxin conversion to carcinogen. Chokeberry juice was found to stimulate the growth of intestinal microorganisms such as *Bifidobacterium*, *Lactobacillus* and *Enterobacteriaceae* bacteria and to decrease the growth of bacteria which belong to *Enterococcus* species [53]. Similarly, incubation of malvidin, delphinidin, petunidin, peonidin, and cyanidin 3-glucosides mixture with faecal bacteria resulted in the increase in the growth of *Bifidobacterium* ssp. and *Lactobacillus* ssp. [54].

Some studies correlate serum anthocyanin concentration after anthocyanin-rich food ingestion with antioxidant capacity [55–58]. The qualitative and quantitative changes in anthocyanins during gastrointestinal digestion of both food matrices tested caused decrease in free radical scavenging capacity and reducing properties (Table 4). Despite the significant increase of anthocyanin content in gastric digestion of whole red cabbage, we did not observe significant increase of the antioxidant capacity. On contrary, the decrease of anthocyanin content (by 87%) during digestion of anthocyanin-rich extract caused the antioxidant capacity decrease only by about 50%. One can suggest that changes in anthocyanins structures strongly influence their antioxidant activity. Unfortunately, there is no comparative data for the antioxidant activity of acylated anthocyanins and simple phenolic compounds. In addition, the native anthocyanins and their degraded products may interact with each other and/or with other red cabbage constituents that may occur. Similarly to our results, Bouayed et al. [59] demonstrated significantly lower reducing capacity (FRAP) and radical-scavenging activity (ABTS) in gastric and intestinal digests of fresh apple. Contrary to our results, total antioxidant capacity (via FRAP and DPPH assays) of fruit juices was significantly enhanced after in vitro digestion [60].

## 5. Conclusion

In this study we examined the effect of in vitro gastrointestinal digestion and incubation with human faecal microflora on the stability and antioxidant capacity of anthocyanins present in raw red cabbage cv. Koda or in its anthocyanin-rich extract. The results demonstrate that food matrix is an important factor influencing the stability of red cabbage acylated anthocyanins subjected to in vitro gastrointestinal digestion. We suggest that in the case of red cabbage (solid food matrix) other vegetable constituents protect the labile anthocyanins from degradation under the physiological conditions simulated. Furthermore, anthocyanins are released from food matrix during gastric digestion and the significant losses of anthocyanins content and antioxidant activity take place in intestine and colon. To the above, after consumption of red cabbage, the intact anthocyanins may exert their beneficial effects in the stomach, while their degraded products and metabolites may act in intestine. Our studies indicate that the use of pure anthocyanins in simulated digestion experiments gives results different than those estimated for complex, solid food matrix, such as fruits and vegetables and their derived foodstuff, and, for this reason, we should carry out such experiments with food as eaten.

## Figures and Tables

**Table 1:** Total phenolics and anthocyanins content in different digestion phases of red cabbage and anthocyanin-rich extract. Data are means ± SD (*n* = 3).

Digestion phase	Total phenolics^1^	Anthocyanins^2^
Raw red cabbage (mg/100 g of cabbage)		
Initial	108.78 ± 7.61^a^	34.28 ± 1.60^a^
Pepsin digestion	136.04 ± 7.71^a^	55.77 ± 1.19^b^
Pancreatin-bile digestion	126.60 ± 18.25^a^	23.21 ± 1.52^c^

Anthocyanin-rich extract (mg/100 mL of extract)		
Initial	36.04 ± 1.24^a^	30.15 ± 1.24^a^
Pepsin digestion	42.12 ± 3.98^a^	30.13 ± 4.89^a^
Pancreatin-bile digestion	54.73 ± 3.56^b^	3.99 ± 0.08^b^

^1^Expressed as gallic acid equivalents; ^2^expressed as cyanidin 3-glucoside equivalents; superscript letters within the same column indicate significant differences among the phenolics and anthocyanins content, *P* ≤ 0.05.

**Table 2:** Tentative identification of anthocyanins peaks in red cabbage and their concentration^1^ in the samples used for digestion process.

Peak no.^2^	*m*/*z* ^3^	Putative identification^4^	Raw cabbage (mg/100 g)	Anthocyanin-rich Extract (mg/100 mL)
1	774	Cy 3-diglucoside-5-glucoside	3.62 ± 0.15	1.34 ± 0.02
2 2	981 1143	Cy 3-(sinapoyl)diglucoside-5-glucoside Cy 3-(glycopyranosyl-sinapoyl)diglucoside-5-glucoside	0.91 ± 0.02	1.36 ± 0.07
3 3	1083 1113	Cy 3-(glycopyranosyl-*p*-coumaroyl)diglucoside-5-glucoside Cy 3-(glycopyranosyl-feruloyl)diglucoside-5-glucoside	2.71 ± 0.08	3.36 ± 0.11
4	1289	Cy 3-(*p*-coumaroyl)(sinapoyl)triglucoside-5-glucoside	1.00 ± 0.01	0.56 ± 0.01
5	1319	Cy 3-(feruloyl)(sinapoyl)triglucoside-5-glucoside	1.20 ± 0.17	1.55 ± 0.08
6 6 6	921 950 981	Cy 3-(*p*-coumaroyl)diglucoside-5-glucoside Cy 3-(feruloyl)diglucoside-5-glucoside Cy 3-(sinapoyl)diglucoside-5-glucoside	13.10 ± 0.38	10.21 ± 0.06
7 7 7	1128 1158 1188	Cy 3-(sinapoyl)(*p*-coumaroyl)diglucoside-5-glucoside Cy 3-(sinapoyl)(feruloyl)diglucoside-5-glucoside Cy 3-(sinapoyl)(sinapoyl)diglucoside-5-glucoside	11.74 ± 0.16	11.77 ± 0.51

^1^Expressed as cyanidin 3-glucoside equivalents; ^2^peak numbers refer to Figure 1;

^3^determined by MALDI-TOF MS analysis; ^4^identification by comparison with previously reported data [18–20, 22]; Cy: cyanidin.

**Table 3:** Change in the number of faecal microbiota in the course of digestion of red cabbage and anthocyanin-rich extract.

Group of microorganisms Log CFU/g* (±SD)	Control	Red cabbage extract	Anthocyanin-rich extract
*Lactobacillus* sp.	6.2 ± 0.08^a^	4.7 ± 0.05^b^	5.5 ± 0.20^b^
*Bifidobacterium* sp.	5.4 ± 0.36^ab^	4.7 ± 0.05^a^	5.4 ± 0.07^b^
*Clostridium* sp.	6.3 ± 0.04^a^	4.6 ± 0.13^b^	5.7 ± 0.14^c^
*Bacteroides* sp.	7.2 ± 0.13^a^	2.6 ± 0.21^b^	5.5 ± 0.23^b^
*Enterococcus* sp.	5.7 ± 0.09^a^	3.4 ± 0.04^b^	3.6 ± 0.16^b^
*Entertobacteriaceae* family	7.2 ± 0.16^a^	4.8 ± 0.22^b^	5.3 ± 0.29^b^
Total anaerobic bacteria	10.0 ± 0.02^a^	6.5 ± 0.08^b^	6.6 ± 0.05^b^

*Log CFU/g wet weight of feces; data are means ± SD (*n* = 3).

Superscript letters within the same row indicate significant differences among the number of faecal microbiota, *P* ≤ 0.05.

**Table 4:** Antioxidant capacity (*μ*mol TE equivalents/g of red cabbage or per mL of anthocyanin-rich extract) of untreated and digested samples. Data are means ± SD (*n* = 3).

Digestion phase	ABTS	FRAP
Raw red cabbage		
Initial	14.18 ± 0.21^a^	4.91 ± 0.01^a^
Pepsin digestion	13.02 ± 0.60^a^	4.36 ± 0.63^a^
Pancreatin-bile digestion	9.07 ± 0.23^b^	2.93 ± 0.21^b^

Anthocyanin-rich extract		
Initial	4.79 ± 0.23^a^	2.25 ± 0.15^a^
Pepsin digestion	3.57 ± 0.24^a^	2.21 ± 0.19^a^
Pancreatin-bile digestion	2.42 ± 0.14^b^	1.13 ± 0.08^b^

Superscript letters within the same column indicate significant differences among the antioxidant capacity, *P* ≤ 0.05.
